# Lions and Prions and Deer Demise

**DOI:** 10.1371/journal.pone.0004019

**Published:** 2008-12-24

**Authors:** Michael W. Miller, Heather M. Swanson, Lisa L. Wolfe, Fred G. Quartarone, Sherri L. Huwer, Charles H. Southwick, Paul M. Lukacs

**Affiliations:** 1 Colorado Division of Wildlife, Wildlife Research Center, Fort Collins, Colorado, United States of America; 2 City of Boulder Open Space and Mountain Parks, Boulder, Colorado, United States of America; 3 Department of Ecology and Evolutionary Biology, University of Colorado at Boulder, Boulder, Colorado, United States of America; University of Liverpool, United Kingdom

## Abstract

**Background:**

Contagious prion diseases – scrapie of sheep and chronic wasting disease of several species in the deer family – give rise to epidemics that seem capable of compromising host population viability. Despite this prospect, the ecological consequences of prion disease epidemics in natural populations have received little consideration.

**Methodology/Principal Findings:**

Using a cohort study design, we found that prion infection dramatically lowered survival of free-ranging adult (>2-year-old) mule deer (*Odocoileus hemionus*): estimated average life expectancy was 5.2 additional years for uninfected deer but only 1.6 additional years for infected deer. Prion infection also increased nearly fourfold the rate of mountain lions (*Puma concolor*) preying on deer, suggesting that epidemics may alter predator–prey dynamics by facilitating hunting success. Despite selective predation, about one fourth of the adult deer we sampled were infected. High prevalence and low survival of infected deer provided a plausible explanation for the marked decline in this deer population since the 1980s.

**Conclusion:**

Remarkably high infection rates sustained in the face of intense predation show that even seemingly complete ecosystems may offer little resistance to the spread and persistence of contagious prion diseases. Moreover, the depression of infected populations may lead to local imbalances in food webs and nutrient cycling in ecosystems in which deer are important herbivores.

## Introduction

Prion diseases affect several mammalian species worldwide, with notable economic and health implications [1], [2]. Two of the known prion diseases are contagious: scrapie of domestic sheep and goats [3] and chronic wasting disease of several species in the deer family [4], [5]. Chronic wasting disease epidemics occur naturally in some native North American deer (*Odocoileus* spp.), wapiti (*Cervus elaphus nelsoni*), and moose (*Alces alces*) populations [5], [6], but whether prion disease was part of the evolutionary history of these host species or is a newly emerging pathogen remains unknown [4]–[6]. Another lingering uncertainty is the effect of natural prion infection on individual host survival and the cumulative impacts of epidemics on population performance and stability. In the absence of empirical data, such effects have been forecast using models [6]–[8] parameterized from observations of natural and experimental prion infections in captive deer [4], [8]–[10]. Regardless of whether transmission is assumed to be density- or frequency- dependent, unmanaged epidemics are predicted to depress deer populations over the course of several decades. If manifested as predicted, the impacts of prion disease epidemics would extend not only to the health and stability of affected host populations, but also perhaps to the health and stability of ecosystems where these species are the primary large herbivores and important as prey.

To begin understanding the implications of prion disease epidemics for native ecosystems and food webs, we measured the effects of prion infection on survival of mule deer (*O. hemionus*) residing on Table Mesa and the lower slopes of Green and Bear mountains southwest of Boulder in northcentral Colorado, USA, an area collectively referred to here as “Table Mesa”.

## Results

**Figure 1:**
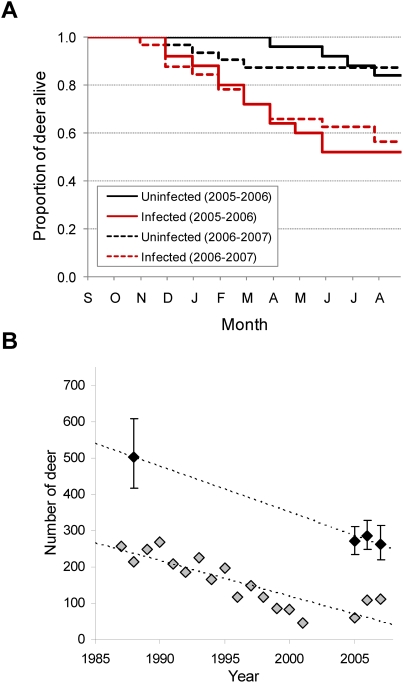
Mule deer survival and population trends at Table Mesa. (A) Survival of prion-infected and uninfected mule deer, 2005–2007. (B) Mule deer population trends, 1987–2007, reflecting declines in both estimated population size (black diamonds, bars ±95% confidence interval) and mean daily counts (gray diamonds) that coincided with emergence of prion disease during the same period; 1987–2001 data provided by the City of Boulder.

Annual survival of prion-infected adult (>2 year old) deer (0.53, 95% binomial confidence interval [CI] 0.39–0.66; n = 57) was markedly lower than survival of uninfected deer (0.82, 95% binomial CI 0.70–0.91; n = 57) (χ^2^ = 9.65, P = 0.0019, hazard ratio = 3.84, 95% CI 1.64–8.99; Table 1) (Fig. 1A). Estimated average life expectancy for infected deer was only an additional 1.6 years, compared to an additional 5.2 years for uninfected deer. Overall survival was similar between sexes (χ^2^ = 0.07, P = 0.79; Table 1). Nearly 75% of the infected deer that died did so during the winter months (November–April) (Fig. 1A).

**Table 1:** Model selection statistics and hazard ratio estimates for influences of prion infection status (“status”) and sex on mule deer survival at Table Mesa.

Model	Model selection				Hazard ratio		
	AIC	ΔAIC	L(model|data)	w_r_	status	sex	sex*status
Status	72.98	0	1.0000	0.654	3.84		
status+sex	74.90	1.926	0.3817	0.25	3.86	1.106	
status+sex+sex*status	76.86	3.882	0.1436	0.094	4.25	1.27	0.832
Sex	84.70	11.72	0.0028	0.002		1.068	

**Figure 2:**
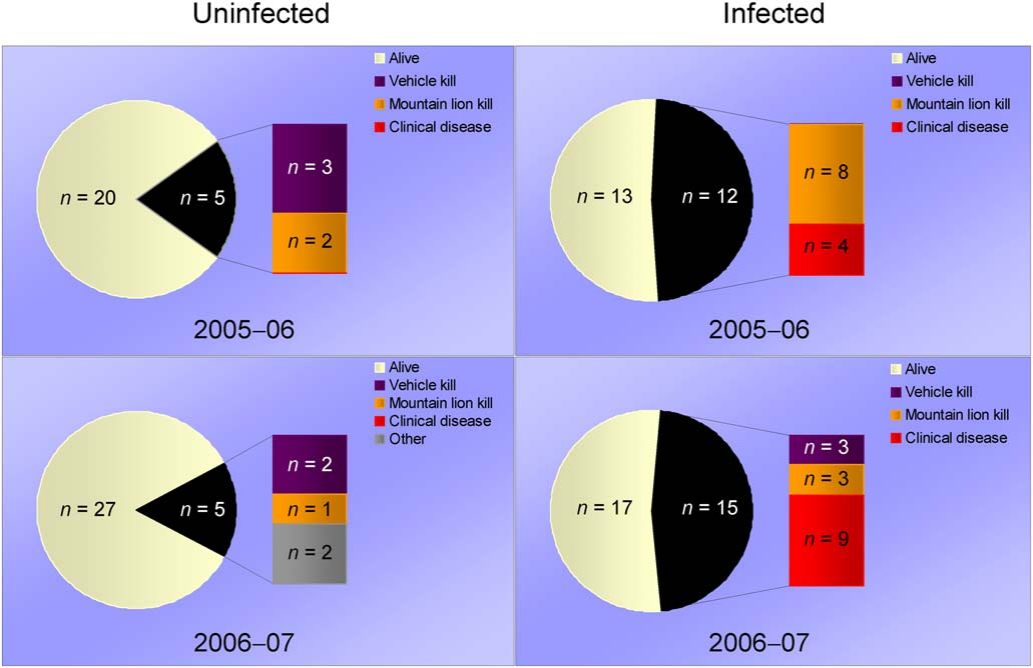
Causes of death in prion-infected and uninfected mule deer at Table Mesa. Mortality was higher among prion-infected deer; only about half of the infected deer survived annually (September–August) in both years (2005–06 and 2006–07).

Thirteen of the 27 infected deer that died apparently succumbed to the “chronic wasting” syndrome [4], [5] and 11 others were killed by mountain lions (*Puma concolor*) (Fig. 2). Few of the deer killed by mountain lions were recorded as noticeably ill by field observers prior to their deaths, suggesting that relatively subtle changes in behavior or condition may have been sufficient cues to draw a predator's attention to infected individuals or increase their vulnerability to attack.

Of 131 different deer captured over the 2-year period, 123 were homozygous for serine at codon 225 of their endogenous prion protein (PrP) gene; the remaining eight (6%) were serine-phenylalanine heterozygotes at codon 225. Overall, 31 (25%; 95% binomial CI 18–34%) serine homozygotes were infected when first sampled; similarly, two (25%; 95% binomial CI 3–65%) serine-phenylalanine heterozygotes also were infected when first sampled.

**Figure 3:**
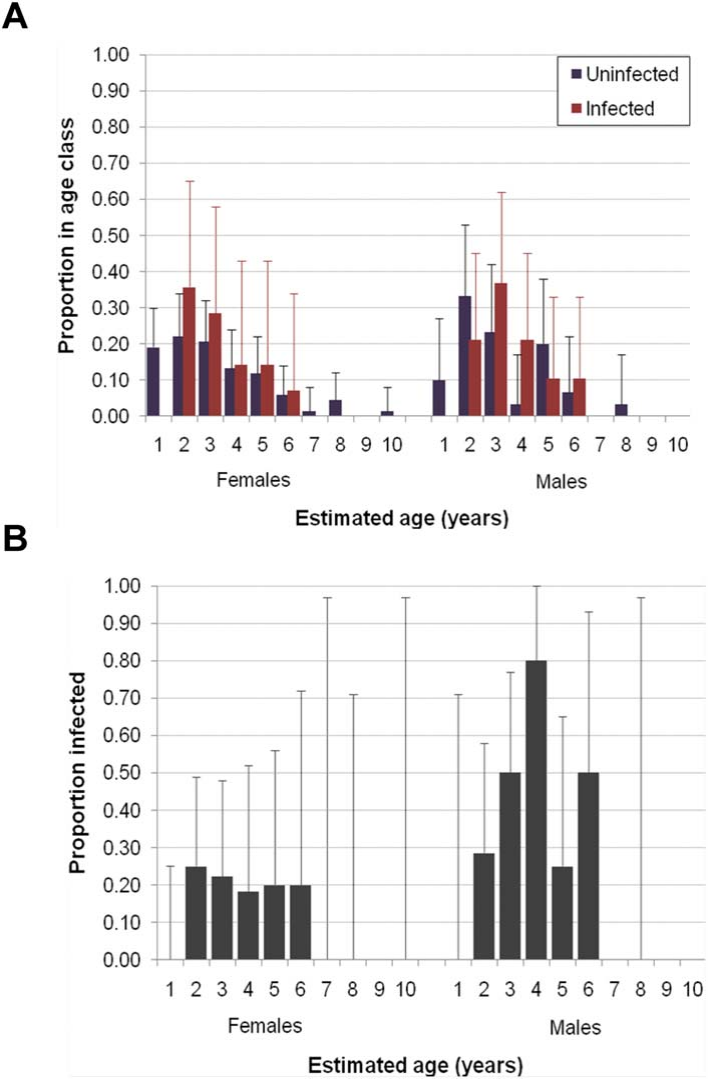
Demographic distribution of prion infection among Table Mesa mule deer. (A) Age distributions of prion-infected male and female deer as compared to age distributions of apparently uninfected female and male deer sampled, expressed as the proportion of the total number of deer in respective sex×infection status group that occurred in each age class. (One-year-old deer likely were underrepresented in our sample because we avoided capturing them for use in survival comparisons.) (B) Age class-specific estimates of prion infection prevalence for sampled female and male deer. Bars are +95% binomial confidence intervals of estimated proportions.

Prevalence among the 46 adult male deer we sampled (41%; 95% binomial CI 27–57%) was about twice prevalence among the 69 adult females (20%; 95% binomial CI 12–32%); infections were not detected in any of 16 deer first sampled when they were <2 years old (Fig. 3). Nearly 80% of the infected deer of both sexes were 2–4 years old (Fig. 3A). Prevalence appeared to vary more across age classes among males than among females (Fig. 3B).

Of 65 initially uninfected deer that we sampled in at least two consecutive years (n = 77 deer×years of presumed susceptibility), 19 became infected (estimated incidence = 0.23 new infections per previously uninfected deer per year; 95% binomial CI 0.15–0.34 new infections per previously uninfected deer per year). As reflected in prevalence trends, incidence tended to be higher in male deer (n = 22 previously uninfected deer×years; estimated incidence = 0.36 new infections per previously uninfected deer per year, 95% binomial CI 0.17–0.59 new infections per previously uninfected deer per year) than in females (n = 59 previously uninfected deer×years; estimated incidence = 0.19 new infections per previously uninfected deer per year, 95% binomial CI 0.1–0.31 new infections per previously uninfected deer per year).

## Discussion

Selectively removing infected individuals from a population should be an effective disease control strategy [7], [11]–[13], but under conditions where predation exacerbates pathogen transmission prevalence can be elevated paradoxically [14]–[16]. At best, selective predation did not appear to be controlling prion transmission at Table Mesa. Although prion-infected deer were much more likely to be killed by mountain lions than uninfected deer (relative risk = 3.67, 95% CI 1.08–12.45), prevalence and incidence of prion infection were still remarkably high: about one fourth of the adult deer in our sample were infected when first captured, and about one fourth of the susceptible adult deer became infected annually. Observed prevalence was relatively high compared to that previously reported in mule deer populations elsewhere [6], [17], but still within the range predicted by a simple model of prion epidemic dynamics [8]. No previous attempts had been made to estimate prevalence or control prion disease in the Table Mesa herd, and this deer population is largely protected from hunting by humans. Consequently, whether our study simply included a period of high prevalence and incidence within the course of a typical epidemic in a mule deer herd or represents an atypical situation cannot be discerned. Regardless, our data show that prion infection in a natural population can surge seemingly unabated even in the face of intense selective predation.

If sustained, the combination of high prevalence and low survival among infected individuals portend adversity for a mule deer population. Average adult survival in a prion-infected deer herd can be calculated as
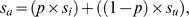
where *s_a_* is overall adult deer survival, *s_i_* is survival of infected deer, *s_u_* is survival of uninfected deer, and *p* is prevalence of prion infection. Although survival among uninfected deer approximated the range-wide average for adult mule deer [18], estimated overall adult survival for the Table Mesa deer herd was 0.72. A mule deer population would be expected to decline in the face of such rates [18], and observed trends in deer abundance at Table Mesa appear consistent with these predictions. Based on historical field records and observations, prion disease likely has been occurring in this area since at least 1985, and its emergence over the last two decades or more has coincided with a 45% decline in estimated deer abundance despite ample habitat and protection from hunting by humans (Fig. 1B).

In addition to the direct effects of disease on deer survival, the epidemic at Table Mesa could be producing an abundance of vulnerable prey [11], [12], [19], [20], thereby enhancing mountain lion survival and reproduction. Behavioral changes including reduced alertness in the later stages of chronic wasting disease [4], [5] should enhance predator success [11], [20]. Although local mountain lion abundance has not been estimated, at least eleven individuals resided in or within ∼7 km of the Table Mesa area sometime during the course of our study. Whether mountain lion predation somehow reciprocally enhances prion transmission is unknown. However, the tendency for predation to promote social grouping among herbivores [21] could help sustain transmission by maintaining relatively high effective densities [14] even as overall deer abundance declines. One possible outcome of such interplay would be the local depression of deer and concurrent overabundance of mountain lions relative to their preferred natural prey.

Our findings provide compelling evidence that prion epidemics can affect mule deer population dynamics locally – whether or not native ecosystems dominated by deer species will be altered at larger geographic scales by the emergence of contagious prion disease remains to be determined.

## Materials and Methods

### Study area

The ∼23 km^2^ “Table Mesa” study area in Boulder, Colorado, USA included low-elevation (1,660–2,050 m) mule deer range at an urban-open space interface including private and public lands (City of Boulder Open Space and Mesa Parks) bounded by Baseline Road on the north, Colorado Highway 93 on the east, South Boulder Creek on the south, and the Flatiron Mountain front to the west. Our study area encompassed native habitats and urban landscapes developed within those habitats, best characterized as mountain shrub habitat interspersed with mixed forb and grassland openings and timbered patches dominated by ponderosa pine (*Pinus ponderosa*). Mule deer lived throughout the study area, often close to human dwellings.

### Study design

We compared annual (September–August) survival of prion-infected and apparently uninfected adult mule deer using a cohort design. We captured, tonsil biopsied [22], and permanently marked 131 different deer over a 2-year period. In 2005, 80 adult deer were captured and tested. In 2006, we captured and tested 79 adult deer, including 44 individuals that were test-negative in 2005; we did not recapture tonsil biopsy-positive deer. (In 2007, we also recaptured and tested 37 surviving individuals that were test-negative in 2006 to determine their final infection status.) Although we tried to avoid capturing subadult (<2-year-old) deer, we inadvertently captured 16 individuals that were <2 years old at the time of capture; these subadults were not used for survival comparisons because infection rates typically are low in this age class [6], [17].

For each year, each tonsil biopsy positive deer was matched by sex, age, PrP genotype [23], and capture location (as a proxy for home range) with a biopsy negative deer; pairings for 2005–06 and 2006–07 were independent. In all, we monitored, located, and observed 57 pairs of prion infected–uninfected deer (25 pairs in 2005–06, 32 in 2006–07), and investigated causes of death. Regardless of infection status, we allowed deer to survive until their natural demise without intervention (except where individuals were judged to be “terminal” and euthanized for humane reasons, as detailed below).

### Field procedures

Our field procedures were reviewed and approved by the Colorado Division of Wildlife Animal Care and Use Committee (CDOW ACUC; file 08-2005) and generally followed those developed elsewhere [13], [22]. All deer were captured and handled during September–February using previously-approved methods (CDOW ACUC files 12-1999, 07-2001, 07-2002, 05-2003).

We used tonsil immunohistochemistry (IHC), a sensitive and specific test for prion infection in mule deer that detects a high proportion of the infected subpopulation [10], [22], to classify deer as infected or uninfected. We regarded deer with abnormal prion accumulation in ≥1 lymphoid follicle as infected with prion disease; individuals with no evidence of prion accumulation were regarded as uninfected at the time of sampling. We anesthetized all deer prior to sampling. Biopsies were performed by veterinarians, biologists, or technicians with previous training and experience in these procedures; methods were as described elsewhere [13], [22]. We estimated ages of deer by dental replacement and wear patterns. All sampled deer received a single antibiotic dose (florfenicol; 40 mg/kg, subcutaneously). In conjunction with biopsies, we collected up to 50 ml of blood via jugular or cephalic venipuncture.

We marked all sampled deer with individually-identifiable plastic ear tags and with collars equipped with mortality-sensing VHF radio transmitters prior to recovery and release. Residual sedation or anesthesia was antagonized to speed recovery.

Marked deer were tracked twice weekly via telemetry to monitor survival and movements. Deer whose telemetry devices transmitted a mortality signal were tracked down immediately. Sites where study deer died were marked using a global positioning system (GPS) unit, examined, and photographed. The probable cause of death was recorded based on established site evaluation procedures. Whenever feasible, deer carcasses were recovered for necropsy or examined in the field to determine cause of death and tissue samples (retropharyngeal lymph node and medulla oblongata at the obex) collected. Deer carcasses that were cached and showed evidence that trauma and localized hemorrhage had occurred in musculoskeletal tissue prior to death were classified in the field as mountain lion kills and were not removed from the location where they were found. We used IHC of lymph node and medulla to confirm prion infection and assess disease progression [10], [24].

Geographic locations were determined on all marked deer at least once every 15 days (≥24 locations/year) to estimate home range and describe movement patterns. Locations were based on visual observation. In addition, deer were observed and body condition assessed visually at least monthly.

In addition to collecting survival and spatial data, we used marked deer to conduct annual, ground-based mark-resight inventories [25], [26] to estimate herd size and composition, as well as annual productivity. For these inventories, established routes were walked or driven daily during late November–early December until a target observation quota of ≥1,000 deer (including all repeated observations) was reached; this quota was based on the estimated sample needed to achieve a 10% coefficient of variation on the population estimate. Observers recorded the total number of ≥1 year old male deer, ≥1 year old female deer, and <1 year old deer in each group encountered, as well as the unique collar identification number for each marked deer observed; the approximate geographic location of the group also was estimated based on a hand-held GPS unit.

To assess long-term trends in deer abundance, we also reviewed and assembled original field data from unpublished mark-resight inventories and systematic counts conducted by the City of Boulder in the Table Mesa area in 1985–2001; although daily count data for routes corresponding to our study area were available for 1987–2001, we were only able to gather all of the information needed for a reliable mark-resight estimate of deer abundance for the data from 1988. We also examined the trend in mean of daily counts for each year as a proxy for trends in deer abundance because count data were available and consistently recorded for almost every year since 1987.

### Endpoints

Regardless of infection status, we allowed marked deer to survive until their natural demise without intervention except as described. Once deer were enrolled in the study, they were not euthanized unless they became severely debilitated as a result of prion infection or other health problems. Field assistants blinded to infection status of individual deer observed all marked deer about once a month to assess health. Signs of prion disease [4], [5], [10] were subjectively scored (0 = not shown, 1 = subtle, 2 = obvious) for behavioral changes, loss of body condition, stumbling or lack of coordination, inability to rise or retreat from a threat, and salivation, regurgitation, or excess drinking, and a total score calculated (maximum clinical score = 14). Deer with clinical scores ≥10 were regarded as being in “end-stage disease” (prion or other) and euthanized.

Because this study was conducted partially in an urban area, deer severely injured during the course of the study (e.g., from collisions with vehicles) were euthanized on a case-by-case basis consistent with established operating policies and agency practices (e.g., darting and lethal injection, gunshot); in these cases, the cause of death was recorded as “trauma–euthanized”, and these deer were not censored from survival calculations.

### Analysis

We tabulated test-positive and -negative results annually and used them to estimate prevalence and incidence of prion infection. We compared survival of prion-infected and uninfected mule deer using matched case-control conditional logistic regression [27] in SAS 9.1; model selection was based on Akaike's Information Criterion (AIC) [28]. Individual covariates included infection status, sex, and age. We also used PrP genotype and capture location in grouping deer, but did not use these covariates in our analyses; instead, we paired pairs of male and female deer by capture location and genotype and then used these groups of four individuals (n = 12 groups in 2005–06, 16 groups in 2006–07) as the sample unit for our analyses to account for other potential influences on survival. We estimated relative risks of specific causes of mortality (predation, vehicle collisions) for prion-infected mule deer as compared to uninfected individuals. For all comparisons, we used ΔAIC>2.0 [28] or α = 0.05 in ascribing significance to results of analyses.
